# Dataset from a proteomics analysis of tumor antigens shared between an allogenic tumor cell lysate vaccine and pancreatic tumor tissue.

**DOI:** 10.1016/j.dib.2022.108490

**Published:** 2022-07-25

**Authors:** C. Stingl, S.P. Lau, S.H. van der Burg, J.G. Aerts, C.H.J. van Eijck, T.M. Luider

**Affiliations:** a Department of Neurology, Clinical and Cancer Proteomics, Erasmus University Medical Center, ‘s-Gravendijkwal 230, Rotterdam 3015CE, the Netherlands; b Department of Surgery, Erasmus University Medical Center, ‘s-Gravendijkwal 230, Rotterdam 3015CE, the Netherlands; c Department of Pulmonary Medicine, Erasmus University Medical Center, ‘s-Gravendijkwal 230, Rotterdam 3015CE, the Netherlands; d Department of Medical Oncology, Oncode Institute, Leiden University Medical Center, P.O. Box 9600, Leiden 2300RC, the Netherlands; e Erasmus MC Cancer Institute, Erasmus University Medical Center, ‘s-Gravendijkwal 230, Rotterdam 3015CE, the Netherlands

**Keywords:** Tissue proteomics, Deep proteome analysis, Tandem mass tag (TMT) labelling, Multi-dimensional protein identification technology (MudPIT), High-field asymmetric waveform ion mobility spectrometry (FAIMS), Liquid chromatography coupled to mass spectrometry (LC-MS)

## Abstract

The data described was acquired as part of a clinical study with the aim to investigate the potential of tumor-reactive T-cell response as response to vaccination of pancreatic cancer patients with an allogenic tumor cell lysate vaccine (Lau et al., 2022). Proteomics analysis was carried out to identify tumor antigens that are shared between the allogeneic tumor cell lysate used for the vaccine and pancreatic ductal adenocarcinoma (PDAC) tissue samples. To this objective, cell lysates of the vaccine and of nine tissue samples were enzymatically digested and isotopically labeled with tandem mass tags (TMT) in a so-called six-plex manner (Thermo Fisher Scientific). Three pools were prepared by mixing the samples according to their TMT-labels. Subsequently, the three sample pools were fractionated into 24 fractions with high-pH reversed phase chromatography. These fractions were first analyzed on a nano-liquid chromatography (LC) system online coupled to a high-resolution Eclipse Orbitrap mass spectrometer (MS) equipped with a high-field asymmetric-waveform ion-mobility spectrometry (FAIMS) source using a data-dependent MS2 shotgun method. Overall, 126,618 unique peptide sequences, on basis of 768,638 peptide spectra matches and corresponding to 7,597 protein groups, were identified in the total sample set including 61 tumor antigens (Supplement Table S2 in Lau et al. 2022) that were prioritized by Cheever and co-workers as vaccine target antigens on basis of a series of objective criteria (Cheever et al., 2009). In the second phase of the experiment, this set of tumor antigens was targeted using a serial precursor selection (SPS) MS3 method. From this data, ion trap MS2 and Orbitrap MS3 fragment spectra were extracted for peptide identification (protein sequence database-dependent search) and relative quantification using the TMT labels, respectively. The dataset ultimately allowed the identification and quantification of 51 proteins and 163 related peptide precursors with the TMT labels (see Fig. 2B and Supplemental Fig. 8, Lau et al. 2022).

## Specifications Table

**Table:** 

Subject	Omics: Proteomics (Biological sciences) and Oncology (Health and medical sciences)
Specific subject area	Targeted proteomic analysis to determine antigens shared between pancreatic ductal adenocarcinoma and an allogenic cell lysate vaccine combining TMT labelling, high-pH preparative LC, and FAIMS Eclipse Orbitrap MS.
Type of data	TableFigure
How the data were acquired	Two-dimensional chromatography with high pH and reverse phase chromatography (Ultimate 3000 and preparative fractionation).Ultimate 3000 nano RSLC (Thermo Fisher Scientific, Germering, Germany) coupled to an Orbitrap Eclipse Tribrid Mass Spectrometer equipped with a High Field Asymmetric Waveform Ion Mobility Spectrometry (FAIMS) interface (Thermo Fisher Scientific, San Jose, CA, USA).
Data format	Raw and Analyzed
Description of data collection	Data-dependent ion trap MS2 shotgun method for deep proteome analysis (PXD032800) and targeted SPS-MS3 (Orbitrap) method for the targeted quantitative analysis (PXD025210) on selected tumor antigen candidates.
Data source location	Erasmus University Medical Center, Department of Neurology, Rotterdam, The Netherlands
Data accessibility	All MS data (raw), including detailed information about the targeted method, fragment spectra (mgf) used for database search and TMT quantification, and results of database search (mzident) have been made public available via ProteomeXchange with the following identifiers PXD025210 (https://www.ebi.ac.uk/pride/archive/projects/PXD025210) and PXD032800 (https://www.ebi.ac.uk/pride/archive/projects/PXD032800) [3].
Related research article	Lau, S. P., L. Klaase, M. Vink, J. Dumas, K. Bezemer, A. van Krimpen, R. van der Breggen, L. V Wismans, M. Doukas, W. de Koning, A. P. Stubbs, D. A. M. Mustafa, H. Vroman, R. Stadhouders, J. B. Nunes, C. Stingl, N. F. C. C. de Miranda, T. M. Luider, S. H. van der Burg, J. G. Aerts, and C. H. J. van Eijck. 2022. “Autologous Dendritic Cells Pulsed with Allogeneic Tumour Cell Lysate Induce Tumour-Reactive T-Cell Responses in Patients with Pancreatic Cancer: A Phase I Study.” European Journal of Cancer 169:20–31.

## Value of the Data

• The data provides a comprehensive compilation of TMT-labelled peptides identified in cell lysate and tumor tissue (*N* = 9) with mass spectrometry and is additionally annotated with chromatographic measures under low and high-pH reversed phase chromatography conditions (retention times and fraction numbers, respectively) and FAIMS ion mobility information (individual compensation voltage ranges).
• The dataset can be beneficial for further development of liquid chromatography and mass spectrometry methods for identification and quantification, such as the selection of peptides suitable for targeted measurements (e.g., PRM) or fractionation strategies for deep-proteome profiling.
• The dataset may serve as basis (trainings set) for *in-silico* studies of modelling and predicting peptide properties and behaviors.
• The dataset provides information about peptides of tumor antigen candidates [1,2] that were quantified with a data-dependent shotgun method using pre-defined inclusions list of peptide precursors and peptide-fragments for SPS-MS3 TMT quantification.
• The data can be used for further development of analysis and acquisition software to analyze SPS-MS3 spectra and FAIMS data.

## 1 Data Description

**Fig. 1:**
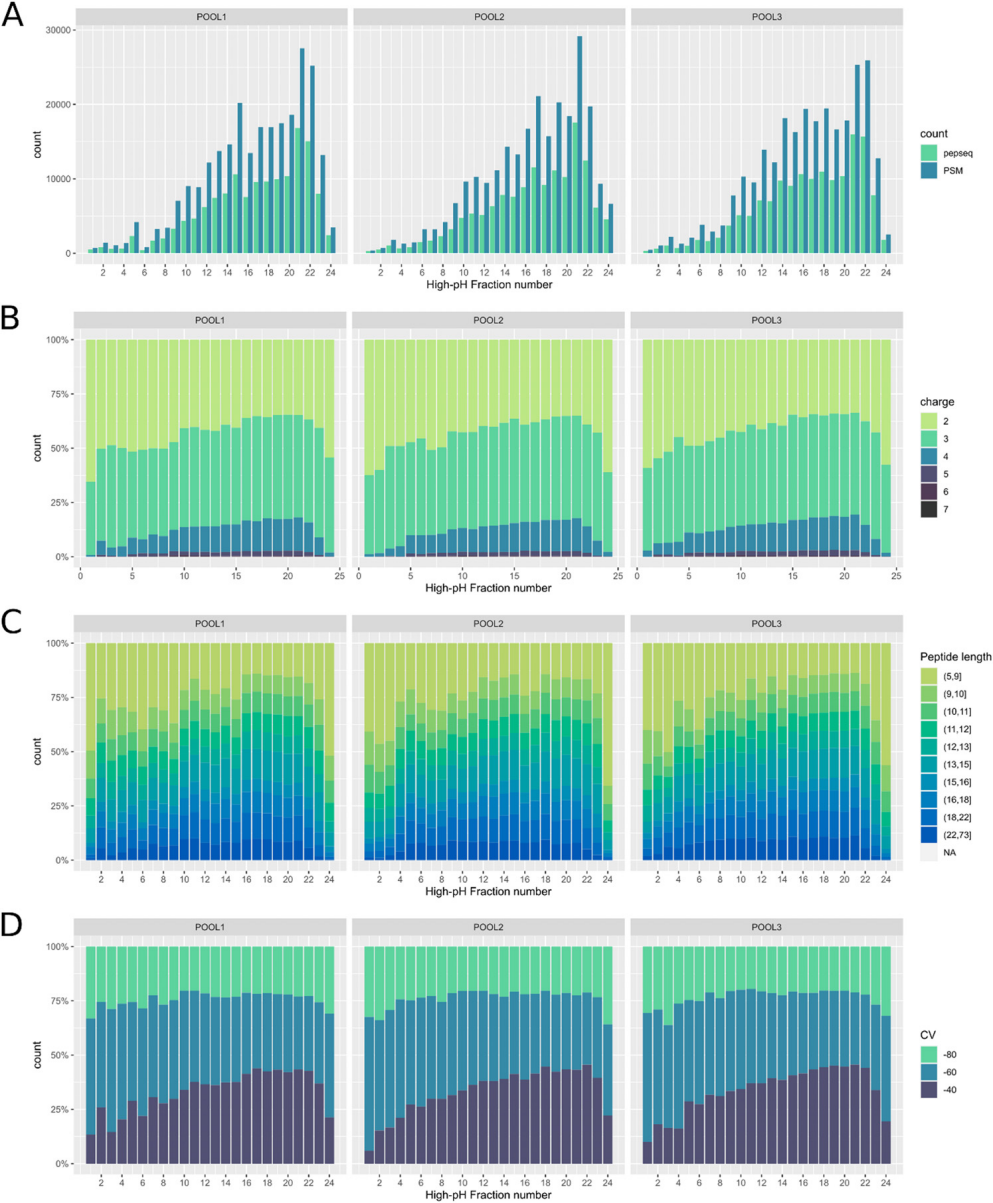
Bar-chart of number of peptide-spectra-matches (PSM) and unique peptide sequences identified in 24 fractions of pool 1, 2, and 3 (A). Distribution of peptide charges (B), peptide length (C) and FAIMS compensation voltage (D) plotted per fraction and pool.

We describe two datasets that were acquired in order to detect and quantify shared tumor antigens between an allogeneic tumor cell line lysate (PheraLys) and tissue samples from pancreatic ductal adenocarcinoma (PDAC) of 9 patients as described by Lau and colleagues [1]. Prior to the LC-MS measurements, all samples were enzymatically digested with trypsin, TMT -labelled, pooled in six-plex, and fractionated into 24 fractions with high-pH reversed phase preparative chromatography. Hence, basis for the LC-MS measurements was a set of 72 analytical samples (24 fractions of 3 pools). The first dataset (ProteomeXchange ID PXD032800) contains spectra of data-dependent shotgun measurements that were set up to identify as many peptides and proteins as possible. To that aim, MS/MS scans were detected in the ion-trap for highest scan speed and sensitivity. In total, these measurements yielded 768,638 peptide-spectra-matches, 126,618 unique peptide sequences and 7,597 proteins groups (protein FDR < 1% and peptide FDR < 0.1%). A complete list of all peptide-spectra-matches identified, annotated with FAIMS compensation voltages and LC retention time and fraction numbers, respectively, is publicly available on ProteomeXchange repository PXD032800. Column characteristics are described in Table 1. Corresponding to this, the numbers of identification per individual fraction and related distributions of peptide charges, peptide length and FAIMS CV fractions are shown in Fig. 1.

**Table 1:** Column names and description used in peptide-spectra-matches tables uploaded to ProteomeXchange repository PXD032800.

Column Name	Description
sample	Name of the analytical samples, composed of pool number and fraction number; (e.g., POOL1-f01 means fraction 1 of pool 1).
file	Raw file name, as uploaded to the repository.
scan	Scan index of raw file.
pepseq	Peptide sequence; lower case letters specify the following modifications: m = oxidation of methionine, c = carbamidomethylation of cysteine, k = TMT-labelled Lysine, any N-terminal amino acid = TMT-labelling of N-terminus.
score	Mascot score.
prec_mz	m/z ratio of peptide precursor.
charge	Charge state of peptide precursor.
rt	Retention time of MS/MS spectra
CV	FAIMS compensation voltage of MS scan.
genes	Gene identifier related to peptide (derived from accession number reported in database search result).
accnrs	Protein accession number related to peptide (Uniprot/Swissprot format).

The second dataset (PXD025210) was acquired for peptide quantification using TMT reporter ions. For this purpose, we applied a serial-precursor selection (SPS) Orbitrap MS3 method in order to reduce non-specific background signal that potentially generates interferences and leads to biased quantifications [4,5]. To compensate for reduced scan speed and sensitivity of the Orbitrap SPS-MS3 method compared to the ion trap MS2 method, we restricted the data-dependent shotgun method using a precursor inclusion list that just allowed MS2 and MS3 precursor selection of predefined peptide precursors and fragments, respectively.

## 2 Experimental Design, Materials and Methods

### 2.1 Sample Collection

All samples used in this work were collected as part of the REACtiVe (Rotterdam pancrEAtic Cancer Vaccination) Trial a single-centre, non-randomized, open-label safety phase I study for patients aged 18 years or older with surgically resected and histologically proven PDAC who have completed standard-of-care treatment. The study was approved by the Central Committee on Research involving Human Subjects (NL67169.000.18) and enrolled in the Dutch trial register (registration number NL7432, https://www.trialregister.nl). Further description about the study, patient inclusion criteria and sample collection were described in detail by Lau and co-workers [1]. In brief, PDAC tissue samples were obtained during surgery (pancreaticoduodenectomy or distal pancreatectomy and splenectomy) and patients were included in retrospect into the study when the diagnosis was confirmed by pathological review and fresh-frozen tumor material was available. Tumor lysates were prepared by manual cutting of the tissue samples into smaller fragments and bead-mediated homogenization in 1 mL Milli-Q for four cycles of 3 min [1]. A Bradford assay was performed to quantify the protein concentration of the tumor lysates before they were aliquoted and stored below -70°C until further analysis. Allogeneic tumor cell lysates (vaccine) were prepared under GMP-compliant conditions and consists of five unique, clinical-grade human multiple myeloma cell lines, which are patented (patent ID P6038325NL). In short, the five cell lines were individually cultured and frozen as bulk products. These bulk products were pooled in equal amounts to form a pre-lysate. The pre-lysate was freeze-thawed multiple times and gamma irradiated to ensure tumor cell death. The formed whole cell line lysate was QC checked, aseptic filled in vials containing 1.05 mL of end product, and stored below -70 °C until further analysis.

### 2.2 Sample Digest and Labelling

Volumes corresponding to 100 µg protein amount (Bradford assay) were dissolved in 2% Sodium deoxycholate (SDC), 100 mM Triethylammonium bicarbonate (TEAB), and 10 mM 1,4-Dithiothreitol and filled up with water to a total of 229 µL. Samples were heated for 2 min at 95°C, intensively sonicated (Branson cup sonification device) for 2 min at 70% intensity, and incubated for a further 30 min at 56°C and mildly shaked. Then, samples were loaded in an Amicon Ultra-0.5 Centrifugal Filter Unit with an Ultracel-30 regenerated cellulose membrane and washed three times with 0.5% SDC, 5% acetonitrile (ACN), and 100 mM TEAB. Next, 15 mM Iodoacetamide was added, samples were incubated at darkness for 30 min and then washed twice with 0.5% SDC, 5% ACN and 100 mM TEAB, and additionally washed twice with 200 mM TEAB. For sample digestion, 4 µg trypsin (Trypsin Gold, Promega) was added and samples were incubated at 37°C over-night (approx. 14 h). Finally, the digest solution was spun down and a small aliquot of 1.8 µL was acidified and diluted (10x), centrifuged and used for nano-LC test runs, during which UV absorbance was detected to determine total peptide abundance. For peptide labeling with Tandem Mass Tags (TMT), a normalized volume corresponding to 30 µg digest was taken and filled up with 200 mM TEAB to a total volume of 75 µL. Then, TMT six-plex reagents were diluted in 100 µL ACN and 31 µL label reagent was added to each samples according to the scheme in Table 2. Samples were incubated for 1 h at 20°C with mild shaking, and thereafter reaction was quenched by the addition of 2.7 µL 5% hydroxylamine. Finally labelled samples were combined into three pools according to the scheme in Table 2, acidified, dried (speedvac concentrator), resuspended in 2% ACN/0.5% TFA and transferred to LC vials for the following preparative fractionation. If not noted otherwise, all reagents were purchased from Sigma-Aldrich/Merck.

**Table 2:** Sample table of three TMT-sixplex pools.

#	Sample ID	Patient	TMT reporter ion	Pool	Category
1	RT002	Pat1	126	POOL1	non-tumor tissue*
2	RT002	Pat1	127	POOL1	tumor lysate
3	RT003	Pat2	128	POOL1	non-tumor tissue*
4	RT003	Pat2	129	POOL1	tumor lysate
5	RT004	Pat3	130	POOL1	tumor lysate
6	Drug product	(vaccine)	131	POOL1	Drug product
7	RT004	Pat3	126	POOL2	non-tumor tissue*
8	RT006	Pat4	127	POOL2	tumor lysate
9	RT007	Pat5	128	POOL2	tumor lysate
10	Drug product	(vaccine)	129	POOL2	Drug product
11	RT008	Pat6	130	POOL2	non-tumor tissue*
12	RT008	Pat6	131	POOL2	tumor lysate
13	Drug product	(vaccine)	126	POOL3	Replicate
14	Drug product	(vaccine)	127	POOL3	Drug product
15	RT010	Pat7	128	POOL3	tumor lysate
16	RT011	Pat8	129	POOL3	non-tumor tissue*
17	RT011	Pat8	130	POOL3	tumor lysate
18	RT012	Pat9	131	POOL3	tumor lysate

⁎ non-tumor tissue: not applicable for this study.

### 2.3 High-pH Reversed Phase Fractionation

Preparative chromatography was conducted on an Ultimate 3000 LC system (Thermo Fisher Scientific) equipped with C18 reversed phase column (Kinetex EVO, 2.1 mm x 150 mm, PN 00F-4725-AN, Phenomenex) operated at an oven temperature of 40°C. Peptides were separated by a binary gradient from 4% to 38% solvent B in 8 min at a flow rate of 450 µL/min, whereby solvent A was composed of 10 mM ammonium formate buffer pH 10 and solvent B was 80% ACN and 10 mM ammonium formate pH 10. Twenty-four fractions of 200 µL (collection period of 26 s) were collected in a 96 well-plate (PN P-96-450V-C, Axygen/Thermo Fisher Scientific), dried (speedvac concentrator), resuspended in 2% ACN/0.1% TFA, split in two aliquots and transferred to a heat-sealed 384 well-plate, where it was stored at 4°C until subsequent LC-MS analysis.

### 2.4 LC-MS Measurements

LC-MS measurements were performed on a nano-LC system (Ultime 3000 RSLC, Thermo Fisher Scientific, Germering, Germany) coupled to an Orbitrap Eclipse Tribrid Mass Spectrometer equipped with a High Field Asymmetric Waveform Ion Mobility Spectrometry (FAIMS) interface (Thermo Fisher Scientific, San Jose, CA, USA). Twenty-four µL peptide fraction were injected and transferred on a trap column (C18 PepMap, 300 µm ID x 5 mm; Thermo Fisher Scientific) using 0.1% trifluoroacetic acid at a flow rate of 20 µl/min, and further eluted and separated on a 50 cm analytical nano-LC column (PepMap C18, 75 µm ID x 500 mm, 2 µm, 100 Å; Thermo Fisher Scientific) using a binary 3 h gradient from 4% to 24% solvent B in 120 min and further increasing to 45% B in 60 min, whereby solvent A was 0.1% formic acid, solvent B 80% acetonitrile and 0.08% formic acid, and a flow rate 300 nL/min and a column temperature of 40°C was applied. For electrospray ionization we used coated silica nano electro-spray emitters (New Objective, Woburn, MA, USA) at a spray voltage of 2.2 kV. FAIMS was setup to collect ion mobility fractions at compensation voltages (CV) of -40, -60 and -80 V. For the untargeted survey experiment (with the aim to identify as many tumor antigens as possible), a data dependent acquisition MS method was used with an Orbitrap survey scan (range 375–1500 m/z, resolution of 120,000, AGC target 400,000), followed by consecutively isolation (isolation with = 0.7 amu), fragmentation (HCD, 35% NCE) and detection (ion trap, AGC 10,000) of the peptide precursors detected in the survey scan until a duty cycle time of 1 s per FAIMS CV fraction was exceeded. Precursor masses that were selected once for MS/MS were excluded for subsequent fragmentation for 60 s. In total, 16 fractions of 3 pools (fractions 6 to 23; 54 runs) were measured with this method. Additionally, four early fractions (fraction 1,2, 5, and 8) and the last fraction (fraction 24) that showed in general comparable low peptide amounts, were measured by a shorter 60 min gradient, with otherwise identical parameters.

Thereafter, a targeted SPS-MS3 methods was developed to specifically acquired MS3 reporter ion spectra with enhanced accuracy and selectivity, whereby a targeted inclusion list for TAG related peptides (MS2 precursor masses) and fragments (MS3 precursors masses; using fragments with relative intensities greater 10%) were prepared on the basis of the results of the preceding survey experiment. The initial list of tumor antigens was derived from a preceding RNA sequencing analysis and literature research [2] and is shown in Lau at al. 2022 (Supplemental Table 2 [1]). Each peptide was specified by the expected m/z ratio, retention time, compensation voltage, and high-pH fraction. For each fraction in which TAG peptides were targeted (fractions 6 to 23), a specifically adapted method (parent mass list table) was prepared containing the expected peptides, but also of the preceding and following fractions to compensate for variation of elution during preparative LC. Up to 10 MS2 fragment masses were allowed for sequential precursor selection (SPS), the MS2 isolation width was 3 m/z (fixed) and HCD collision energy set to 55%. SPS-MS3 scans were acquired in the Orbitrap at a resolution of 50,000, AGC target of 250,000 and maximum injection time of 200 ms. In total we measured 16 fractions of 3 pools with this approach, whereby 2 measurements (fraction 18 and 19 of pool 3) did not yield suitable data because of a hardware failure.

### 2.5 Mass Spectrometry Data Analysis

Acquired RAW data of both, survey experiment and targeted SPS-MS3 runs, were processed with MSaccess and MSconvert (proteowizard version 3.0.19263) [6] to extract scan meta information and fragment ion peak lists as MGF files, respectively. For peptide and protein identification we conducted a Mascot fragment ion database search (v.2.301; Matrix Science) and subsequent carried out determination of the false-discovery rates, protein grouping and exporting of results (spectra report) using the software package Scaffold (version 4.11.1; Proteome Software), whereby following settings were applied: combined human subsets of Swiss-Prot and TrEMBL (download: January 18th 2021, 194,237 entries), decoy search, carbamidomethylation (+57.021 u) of cysteine and TMT6plex labelling (+229) of lysin and the peptide N-terminus as fixed modification, oxidation (+ 15.995 u) of methionine, 10 ppm precursor ion tolerance and 0.5 Da fragment ion tolerance, and 1% peptide and 0.1% protein false discovery rate threshold. Results were exported (via a Scaffold spectra report) from the runs of the survey experiment and compared to the list of TAGs described above to derive a list of targeted TAG peptides for targeted SPS-MS3 measurements. From the targeted SPS-MS3 data, MS3 reporter ions were extracted from the peak lists (mgf files) and assigned to the MS2 spectra identified. The noise level of each MS3 spectra was estimated as the median overall fragment intensity, and for each peptide species and pool the scan with the highest overall reporter ion intensity was selected for quantification. From these scans the ratio of intensity in tissue lysate to PheraLys, the individual intensities and signal to noise ratio (S/N) of the signal were derived for each reporter ion. Peptides were reported as positively detected and quantified in a sample if the S/N of the reporter ion was above 3 and 10, respectively. At a maximum, the five most suitable peptides per proteins were reported and used to calculate quantitative protein properties. Protein intensities were calculated as the sum of peptide intensity normalized by the total number of peptide intensities of reported peptides. For data extraction we used adjusted and in-house written Perl programs [7], and for data analysis and plotting the statistically software package R [8].

## Ethics Statements

The study was approved by the Central Committee on Research involving Human Subjects (NL67169.000.18) as defined by the Medical Research Involving Human Subjects Act. Procedures followed were in accordance with the ethical standards of these committees on human 178 experimentation and with the Helsinki Declaration of 1975, as revised in 2008. The trial is registered with the Netherlands Trial Register, NL7432. An informed written consent was obtained from each subject.

## CRediT authorship contribution statement

**C. Stingl:** Conceptualization, Methodology, Data curation, Visualization, Writing – original draft, Writing – review & editing, Investigation. **S.P. Lau:** Conceptualization, Methodology, Data curation, Visualization, Writing – review & editing, Investigation. **S.H. van der Burg:** Writing – review & editing, Investigation, Supervision. **J.G. Aerts:** Writing – review & editing, Investigation, Supervision. **C.H.J. van Eijck:** Writing – review & editing, Investigation, Supervision. **T.M. Luider:** Writing – review & editing, Investigation, Supervision.

## Declaration of Competing Interest

The authors declare the following financial interests/personal relationships which may be considered as potential competing interests: Please declare any financial interests/personal relationships which may be considered as potential competing interests here: J.G.A: Stock or other Ownership: Amphera. Consulting or Advisory Role: Eli-Lilly, MSD Oncology, Bristol-Myers Squibb, Roche, AstraZeneca. Rest of the authors have no relationship to disclose in relation to the submitted work.

## Data Availability

Autologous dendritic cells pulsed with allogeneic tumor cell lysate induce tumorreactive T-cell responses in pancreatic cancer patients: a phase I study (Reference data) (ProteomeXchange/PRIDE). Proteomics analysis of tumor antigens in allogenic tumor cell lysate and pancreatic tumor tissue (Reference data) (ProteomeXchange/PRIDE).
